# Application of Deep Learning to Predict the Persistence, Bioaccumulation, and Toxicity of Pharmaceuticals

**DOI:** 10.1021/acs.jcim.4c02293

**Published:** 2025-04-03

**Authors:** Dominga Evangelista, Elliot Nelson, Rachael Skyner, Ben Tehan, Mattia Bernetti, Marinella Roberti, Maria Laura Bolognesi, Giovanni Bottegoni

**Affiliations:** † Department of Pharmacy and Biotechnology, Alma Mater Studiorum—University of Bologna, Via Belmeloro 6, Bologna 40126, Italy; ‡ OMass Therapeutics, Building 4000, Chancellor Court, John Smith Dr, Oxford Business Park, ARC, Oxford OX4 2GX, United Kingdom; § Department of Biomolecular Sciences, University of Urbino, Urbino 60129, Italy; ∥ Computational and Chemical Biology, Fondazione Istituto Italiano di Tecnologia, Via Morego 30, Genova 16163, Italy; ⊥ Department of Pharmacy, University of Birmingham, Edgbaston B15 2TT, Birmingham, United Kingdom

## Abstract

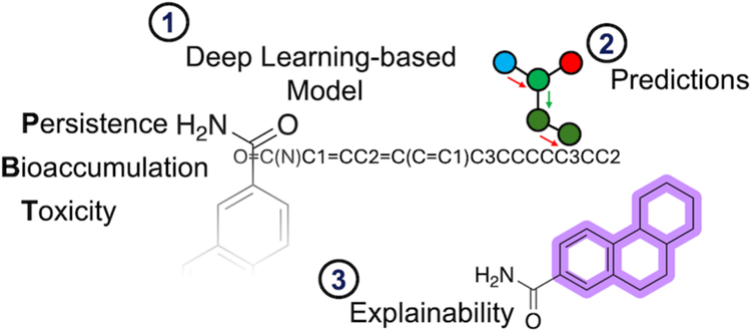

This study investigates the application of a deep learning (DL) model, specifically a message-passing neural network (MPNN) implemented through Chemprop, to predict the persistence, bioaccumulation, and toxicity (PBT) characteristics of compounds, with a focus on pharmaceuticals. We employed a clustering strategy to provide a fair assessment of the model performances. By applying the generated model to a set of pharmaceutically relevant molecules, we aim to highlight potential PBT chemicals and extract PBT-relevant substructures. These substructures can serve as structural flags, alerting drug designers to potential environmental issues from the earliest stages of the drug discovery process. Incorporating these findings into pharmaceutical development workflows is expected to drive significant advancements in creating more environmentally friendly drug candidates while preserving their therapeutic efficacy.

## Introduction

Pharmaceuticals are chemicals with distinctive and rigorous profiles. They are rationally designed to be active, bioavailable, and nontoxic to human patients, endowed with sufficient stability and an appropriate shelf life, thereby ensuring that, upon administration, they reach their intended target in an unaltered, active form. Consequently, in many cases, pharmaceuticals are excreted from the body in their original form or as an active metabolite, ultimately entering and contaminating natural environments and potentially causing a wide range of unintended, harmful effects on the ecosystem. This new awareness of pharmaceuticals as pollutants has prompted calls on the pharmaceutical industry to develop active pharmaceutical ingredients (APIs) that are inherently less toxic for the environment and degrade more rapidly, the so-called “greener APIs”.^1^ Over 600 APIs have been detected in environmental samples, including surface waters and sewage effluents.^2,3^ While the contamination of the aquatic ecosystem occurs through drug excretion and improper disposal, the terrestrial environment is also exposed to APIs through several other means. These include the application of sewage sludge and wastewater, either treated or untreated, to agricultural lands and directly to the excretion of veterinary medicines by animals. All this poses immediate scientific, technological, and regulatory concerns^4,5^ that have prompted proactive toxicity evaluation for environmental risk assessment (ERA) for drugs, prior to their registration.^6^ According to the most recent ERA guidelines for human and veterinary medicines,^7^ an assessment must be performed as early as possible to determine whether a pharmaceutical ingredient is potentially persistent, bioaccumulative, and toxic (PBT), which usually coincides with Phase III clinical trials. Moreover, this assessment should be integrated into the standard drug discovery pipeline,^8^ especially for anti-infectives.^9^ In 2023, the European Chemicals Agency (ECHA) introduced new hazard classifications for PBT and very Persistent and very Bioaccumulative (vPvB) chemicals.^10^ They are now categorized under two crucial hazard classes: “Strongly accumulates in the environment and living organisms including in humans” and “Can cause long-lasting and diffuse contamination of water resources”.^10^ In recent years, machine learning (ML) and deep learning (DL) techniques have significantly enhanced the accuracy and reliability of in silico predictions for molecular properties. A recent bibliometric analysis of cheminformatics/QSAR literature from the 2000 to 2023 period demonstrated the growth in ML applications for chemical property prediction, with publications increasing exponentially over this period.^11^ These computational approaches have been successful in predicting various toxicological end points, from acute toxicity to complex adverse outcomes like drug-induced liver injury (DILI). As reviewed by Fraser et al.,^12^ the intersection of experimental, in silico, and artificial intelligence technologies has created new opportunities to improve this kind of predictions. While traditional toxicity testing heavily relies on animal models that often underperform in predicting human outcomes, ML approaches can integrate diverse structural, physicochemical, and biological data types, possess the ability to identify complex patterns in high-dimensional spaces, and can handle nonlinear relationships between molecular features and toxicity end points. The evolution toward data-driven approaches in environmental pollution research has been further analyzed by Liu et al.,^13^ who highlighted how ML methods have become essential tools for processing complex environmental data sets. This analysis demonstrated that over 40% of environmental ML applications focus on air pollution, while applications in other aspects of environmental science, including toxicity prediction, are still emerging but show great promise.^13^ While the challenge of balancing model complexity with interpretability remains, these computational approaches are particularly significant for addressing emerging environmental challenges, including the aforementioned growing concern about pharmaceuticals as environmental pollutants.

Notably, experimental data on environmental behavior is limited for each individual aspect—persistence (P), bioaccumulation (B), and toxicity (T)—particularly for pharmaceuticals.^14^ The scarcity of experimental data for P, B, and T limits methods for estimating absolute continuous PBT values. In silico screening methods are routinely employed for the identification of potential PBT and/or POP chemicals.^15^ In 2010, Papa and Gramatica developed a compact representation of cumulative PBT behavior called the PBT index.^16^ They created a quantitative structure–property relationship (GP-QSPR) model linking the PBT Index to four simple molecular descriptors using multiple linear regression. The restricted amount of experimental data available for training a QSPR model in the way described by Papa and Gramatica limits the model’s applicability in terms of diversity of the investigated compounds. At the same time, should a regression-based model be retrained on a larger and more heterogeneous data set, accuracy could drop.^17^ Various methods exist for binary classification of chemicals as PBT or non-PBT. Recently, deep learning techniques have been applied to associate chemical properties with these binary classifications. For instance, Sun and colleagues developed a deep convolutional neural network (DCNN) trained for PBT screening, which demonstrated good external prediction accuracy.^18^ While this approach showed promise, it faces challenges inherent to the use of two-dimensional (2D) molecular descriptors. Molecular descriptors with irrelevant physical or chemical meanings often have to be arranged arbitrarily next to each other, potentially introducing uncertainties into the modeling process. This arbitrary arrangement could possibly undermine the scientific significance of the developed model and lead to an inaccurate screening of PBT chemicals. On the other hand, graph neural networks (GNNs) operate directly on molecular structures, offering a more robust approach that preserves chemical and structural information without relying on arbitrary descriptor arrangements. More recently, GNNs have shown promise in predicting various molecular properties, including PBT properties.^19−21^ Unlike traditional approaches, GNNs operate directly on molecular graphs derived from SMILES strings, where atoms are represented as nodes and bonds are represented as edges. This graph-based representation allows for “end-to-end” learning, enabling the model to automatically extract relevant structural features without relying on predefined molecular descriptors. By learning directly from molecular structures, GNNs provide a potentially more robust and interpretable approach to tasks such as PBT classification.

Here, we describe the application of Chemprop,^22^ a message-passing neural network developed by Heid and colleagues, to classify persistence, bioaccumulation, and toxicity (PBT) of compounds. This model is designed to serve as an early screening tool in the drug discovery process, acting as a crucial prefilter to identify potential environmental risks associated with pharmaceutical candidates. By integrating this approach at the initial stages of drug development, we aim to enhance environmental safety and promote sustainable innovation toward greener pharmaceuticals. Starting from PBT classification data, we focused on enhancing the model’s generalizability by monitoring and, ultimately, increasing the dissimilarity between the training and test set molecules by means of a clustering strategy. In addition, we applied the proposed model to a series of molecules of pharmaceutical interest in order to highlight potential PBT chemicals. We also identified PBT-relevant substructures that can act as structural flags, signaling potential environmental issues from the early stages of the drug discovery process. This approach is particularly relevant in light of ECHA’s new hazard classifications, as it allows for proactive measures in pharmaceutical development to carefully avoid or modify substances that might fall into these hazardous categories.^10^

## Methods

**Figure 1:**
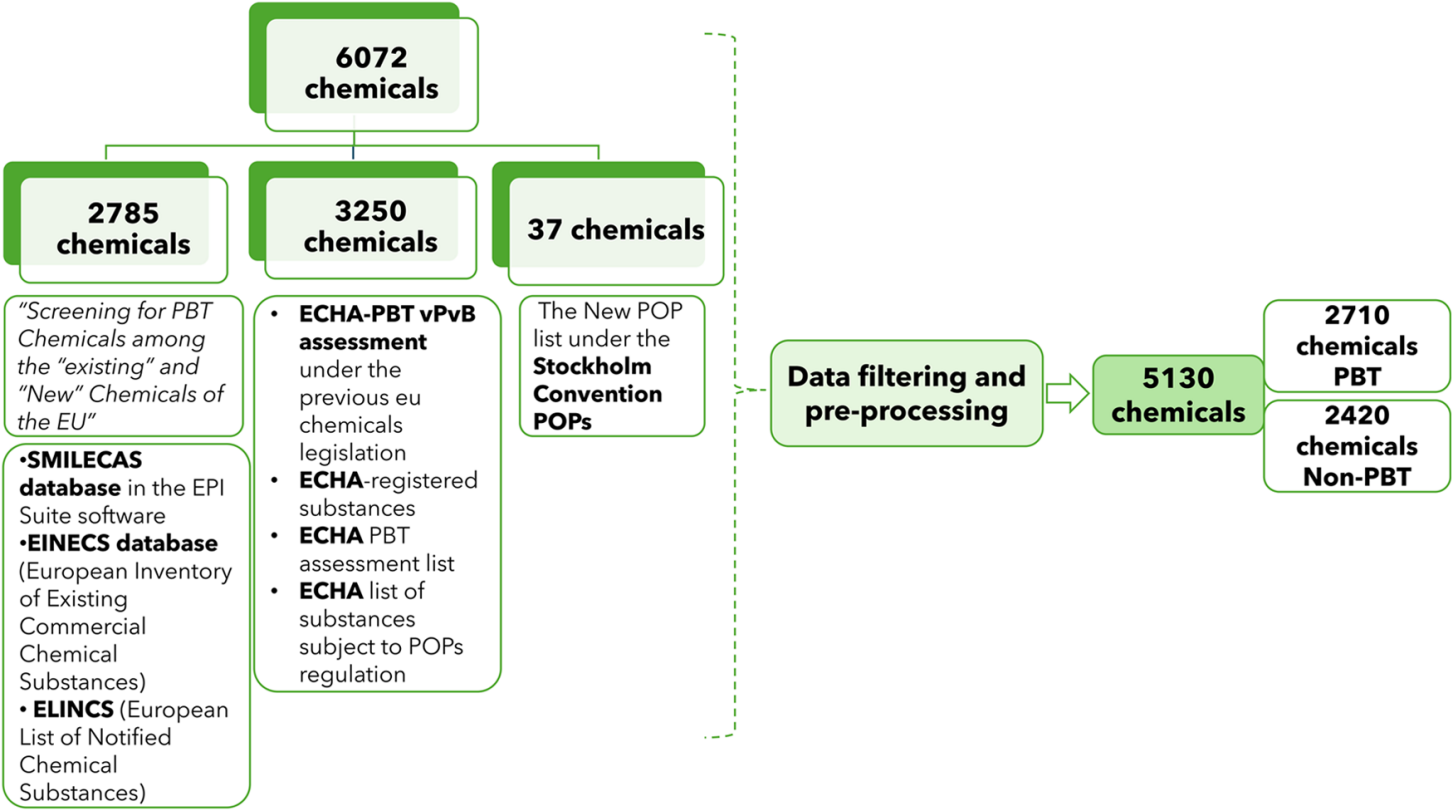
Data set collection and preprocessing: chemicals from European Chemicals Agency ECHA PBT vPvB assessment under the previous EU chemicals legislation,^24^ ECHA-registered substances,^28^ ECHA PBT assessment list,^25^ ECHA list of substances subject to POPs,^26^ Stockholm Convention of POPs,^27^ and European databases SMILECAS,^29^ EINECS,^30^ and ELINCS.^31,32^

In this work, 6072 molecules were initially retrieved (Figure 1): 2970 identified non-persistent organic pollutants (POPs) and 3102 validated persistent bioaccumulative toxic (PBT) or POPs chemicals. The latter group included 2785 potential PBT chemicals identified by Strempel et al.^23^ and 317 expert-verified PBT chemicals from the European Chemicals Agency (ECHA) PBT/vPvB assessments,^24^ the ECHA PBT assessment list,^25^ the ECHA list of substances subject to POP Regulation,^26^ and the new POP list under the Stockholm Convention.^27^ The 2970 Non-PBT chemicals included 2887 compounds from the ECHA-registered substances,^28^ 48 expert-verified compounds gathered from the ECHA PBT/vPvB assessments,^24^ and 35 compounds from the ECHA PBT assessment list.^25^

### Filtering and Preprocessing Procedure

For each data set examined in this study, a standardized procedure for sanitization was employed: when multiple fragments or components were included in the SMILES string, it was assumed that the largest fragment was responsible for the class label, and all molecules in the data set have their charged atoms neutralized, resulting in a data set where each molecule has no formal charges on its atoms and the molecular structure is adjusted to maintain chemical validity. Finally, neutralized molecules were converted to their canonical SMILES representation. Duplicates were removed, together with stereochemistry details. Eventually, the final compiled data set encompassed 5130 chemicals (2710 labeled as PBT and 2420 as non-PBT). The procedure was implemented as an RDKit script (with Python 3.8).^33^

### Splitting Strategies and Statistical Validation

Three strategies for data set splitting were investigated: random splitting (80:20 ratio), clustering splitting, and cluster-centroid splitting using the Butina algorithm.^34^

To validate whether these splitting strategies preserved the independent and identically distributed (IID) assumption, the Kolmogorov–Smirnov (KS)^35^ tests on RDKit fingerprint features were performed for each splitting strategy. The KS test^36^ evaluates whether two samples are drawn from the same underlying distribution by comparing their empirical distribution functions. The null hypothesis (*H*_0_) states that the two samples are drawn from the same underlying distribution. Feature distributions between training and test sets were compared using a significance level of 0.05, with *p*-values above this threshold indicating no evidence to reject *H*_0_, thus strongly hinting at the preserved IID properties. The proportion of fingerprint bits showing significantly different distributions and the mean KS statistic as measures of distribution similarity were also calculated.

### Model Training

Chemprop (v1), a software package that implements a directed-message-passing neural network (D-MPNN) for molecular property prediction, was used in this study to predict the PBT or non-PBT profile of compounds.^22^ A D-MPNN takes input molecules in the SMILES string format and encodes them into molecular graphs using RDKit. Atoms correspond to vertices, and bonds to edges. This kind of neural network processes data in two stages: (i) a message-passing phase, in which information is transferred across the molecule to construct its neural representation, and (ii) a readout phase, which uses the obtained representation to derive specific properties of interest about the molecule. Feature vectors associated with atoms are updated during the message-passing phase, based on their neighbors’ features and the related bonds. The resulting representation of the molecule learned by the model during the first phase can finally be used in the readout phase to perform predictions by means of a feed forward neural network (FFN) that enables the learning of the relation between molecule encoding and specific output properties.^22^ Since the property of interest in our application is a binary PBT classification, the model is trained to output a number between 0 and 1, which represents its prediction about whether the input molecule is PBT or non-PBT, with 1 corresponding to maximum probability of being PBT. Details of the threshold selection analysis for binary classification are reported in the Supporting Information (see Figures S2 and S3 in the SI). Each compound’s graph-based representation is enhanced with 200 molecular features computed using RDKit according to the procedure reported by Yang et al.^37^ This step is performed in order to take into account global properties, which the local-message-passing approach might, by definition, neglect. Additionally, to evaluate the impact of these RDkit molecular descriptors, we also performed D-MPNN training without the 200 RDKit features (Table S2).

The D-MPNN model was used with the default set of parameters: ReLU (Rectified Linear Unit) activation functions; batch size: 50; depth: 3; number of training epochs: 30; ffn_hidden_size: 300; ffn_num_layers: 2.

To evaluate the model’s performance on both the entire data set and specific subsets, the *k*-fold cross-validation method was utilized. The data set was divided into 10 distinct folds. This method generated 10 unique models, each trained, validated, and tested on different data splits, following an 8:1:1 ratio for training, validation, and testing, respectively. For each of the *k* folds, the procedure trains and tests a model on that fold and then aggregates performance metrics across all folds. This approach yields the mean and standard deviation of the model’s performance. Consequently, every data point is used for both training and validation, ensuring each point is used for validation exactly once (see Figures S2 and S3).

### Applicability Domain Analysis

The applicability domain (AD) of the model was assessed using two complementary molecular representations: Morgan fingerprints (radius = 22,048 bits) and RDKit molecular descriptors (200 features). Principal component analysis (PCA)^38^ was employed to reduce dimensionality while retaining 90% of data variance, followed by Mahalanobis distance calculation in the reduced space to determine the AD boundaries.^39^ The AD threshold was set at the 99th percentile of training set distances, using the cluster-centroids data set (*n* = 584) as reference. This approach was applied to evaluate both the test set of singletons (*n* = 1065) and the external data set encompassing pharmaceutical compounds (*n* = 559).^40^

### Performance Metrics and Model Evaluation

The metrics used to evaluate the performance of the proposed model include the receiver-operating characteristic (ROC), accuracy, recall, and specificity. The area under the curve (AUC) quantifies the model’s ability to distinguish between PBT positive and PBT negative labels. It is calculated by plotting the true positive rate (TPR) against the false positive rate (FPR) at various threshold values. True positive rate, or Recall, is defined as
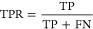
1measuring the fraction of actual PBT positive molecules that were correctly predicted as such.

While the false positive rate as
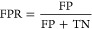
2where TP, TN, FP, and FN represent true positive, true negative, false positive, and false negative instances as predicted by the model, respectively. The AUC is the integral of the ROC curve ranging from 0 to 1.

Accuracy represents the fraction of true results (both true positives and true negatives) with respect to the total number of predictions:
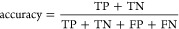
3

Specificity, also called true negative rate (TNR) measures the fraction of PBT negative molecules that were correctly predicted as such:
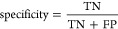
4

The enrichment factor (EF) was used to quantitatively evaluate the contribution of recurring structural patterns in the prediction of PBT behavior:
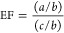
5where *a* is the count of molecules that contain the substructure and that are also PBT, *b* is the total number of PBT in the data set, *c* is the total number of molecules containing the substructure, and *d* is the total number of molecules in the data set (experimental or predicted). An EF of 1 would indicate that the identified substructures are not enriched among PBT predictions compared to the baseline within the entire data set, whereas an EF > 1 would indicate that the substructures identified are over-represented among PBT predictions compared to what would be expected by chance alone within the entire data set. This means that these substructures appear more frequently in PBT compounds than in the general pool of compounds, suggesting a strong correlation between these substructures and PBT positive behavior.

### Model Interpretability and Search for Maximum Common Substructures

Chemprop (v1) implements a built-in “interpret” function to make the interpretability of predictions more straightforward.^37^ Specifically, it employs Monte Carlo tree searches to pinpoint molecular substructures that (i) comprise a minimum of four atoms and (ii) exhibit a minimal PBT prediction score of 0.5. These searches involve iterative selection and pruning of substructures within molecules, aimed at enhancing the prediction accuracy. If the prediction scores for a given compound stabilize at a threshold value of 0.5 or higher, indicating convergence, the Monte Carlo search yields a substructure (the “rationale”) explaining the observed property. Conversely, if convergence does not occur, this signifies that no such explanation could be derived using this approach.^41^ The interpretation run on the trained model was performed with the following parameters: batch size: 500; max_atoms: 20; min_atoms: 4; prop_delta: 0.5; rollout: 20. All identified substructures were standardized and their SMILES notation canonicalized using RDKit,^33^ according to the procedure described above.

## Results and Discussion

### Data set Characterization

In this work, we gathered and examined different data sets of organic molecules. A summary of these data sets is reported in Table 1 (see also the Data Sets section in the SI).

**Table 1:** List of the Data Sets Used in This Work, with Number of Compounds Included and PBT End Point

data set	no. of compound	PBT end point
Papa and Gramatica^16^ (GP-QSPR)	250	P Global Half-Life Index (GHLI), B log BCF, T experimental 96 h LC50
our compiled data set	5130	1 as PBT, 0 as non-PBT
Agrochemicals^42^	1277	none
DrugBank^43^	11168	none
Howard and Miur^15^	277	detected in environmental media
Pharmaceuticals with QSAR PBT^44^	559	1 as PBT, 0 as non-PBT

**Figure 2:**
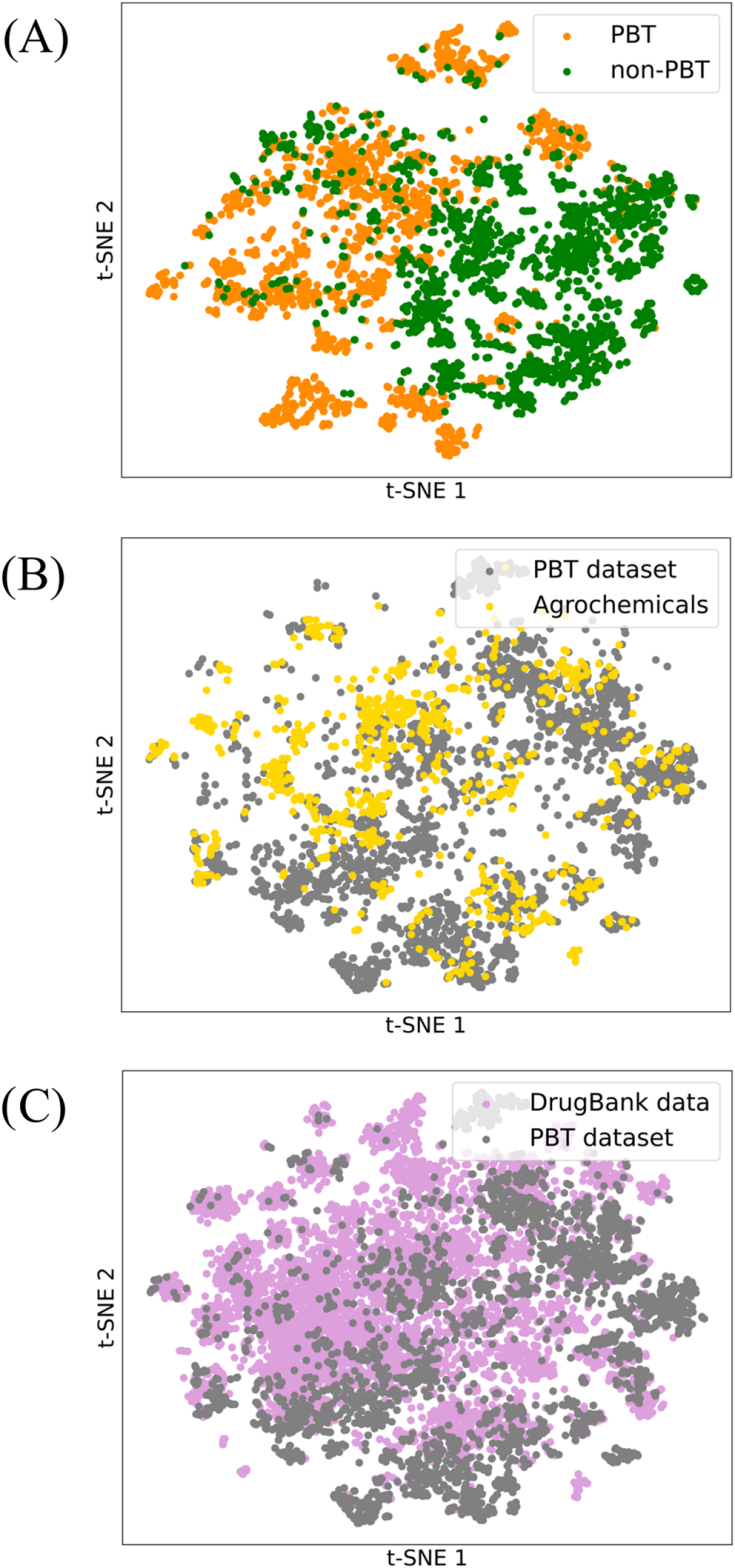
t-SNE plots representing high-dimensional molecular data in two dimensions with 200 RDKit molecular features mapping: (A) the compiled data set, with PBT (orange) and non-PBT (green) chemicals highlighted in different colors, (B) overlap between the compiled (gray) and Agrochemicals^42^ (yellow) data sets, and (C) overlap between the compiled data set (gray) and DrugBank^43^ data (pink) in the defined chemical space.

Experimental data on P, B, and T individually taken are available only for 250 chemicals used to build the GP-QSPR model by Papa and Gramatica^16^ as shown in Table 1. Aiming at gathering a larger and more chemically diverse data set with annotated PBT features, we compiled a binary classification data set from different sources (Figure 1), resulting in 5130 chemicals: 2710 labeled as PBT and 2420 as non-PBT. To this end, we initially applied a standardization procedure to filter and preprocess the collected chemicals (see the Filtering and Preprocessing Proceduresection). We then qualitatively examined the distribution of the compounds in the compiled data set (labeled as PBT and non-PBT) in a chemical space described by the full set of 200 RDKit molecular descriptors^37^ used in the proposed model training and qualitatively investigated the applicability domain using t-Distributed Stochastic Neighbor Embedding (t-SNE) plots (Figure 2, see also SI section t-SNE Dimensionality Reduction Analysis).

t-SNE is a nonlinear dimensionality reduction technique that allows projecting high-dimensional data onto a two-dimensional space for visualization by preserving the local neighborhood information. The employed descriptors encompass a broad range of molecular properties including topological features (like atom and bond counts, molecular connectivity indices), physicochemical properties (such as calculated log *P*, molecular weight, and polar surface area), geometric characteristics (ring counts, stereocenters), and electronic parameters (partial charges, electron distribution), providing a characterization of the molecular space that directly corresponds to the features used in the proposed predictive model.^37^

The t-SNE plot in Figure 2A illustrates how PBT (orange) and non-PBT (green) molecules from our data set mostly occupy distinct regions of the chemical space, yet several areas where the two groups clearly overlap can be identified. This separability suggests how a DL predictive model might be particularly well suited at capturing subtle differences that could escape protocols based on a preconceived list of intuitive features. The t-SNE plots in Figure 2B,C investigate the overlap between the compiled data set (PBT and non-PBT) and two external data sets: Agrochemicals^42^ (yellow circles) and drugs from DrugBank (pink circles). Ideally, for good model generalizability, the compiled data set should significantly overlap in the two-dimensional t-SNE space with the external data sets relevant to the domains of interest, thus suggesting the extent of the model’s applicability. The overlap displayed here suggests that a DL-based model trained on the compiled data set might also make reliable predictions for agrochemicals and drugs. Notably, no clear separation between pharmaceutical compounds from DrugBank and Agrochemicals could be identified (t-SNE plot in Figure S1), with both classes almost homogeneously overlapping throughout the described chemical space. This overlap is noteworthy considering that most compounds experimentally annotated as PBT originate from agricultural and industrial sources, increasing confidence in the idea that a model trained on our data set could be applied to molecules of pharmaceutical interest.

### Data Set Partitioning

After a preliminary evaluation of the potential applicability domain of the model trained on the compiled data set, we considered three strategies for splitting the data into training and test sets: random splitting, clustering-based splitting, and cluster-centroid splitting. The first strategy involved randomly dividing the data set with an 80:20 ratio (train: 4104; test: 1026). In the second approach, the data set was divided into structurally homogeneous groups using the Butina clustering algorithm.^34,45^ Here, the test set only encompassed singletons, i.e., molecules that were the only member of their clusters; all other, nonsingleton molecules were included in the training set (train: 4064 chemicals, test: 1065). The idea was to test for generalizability and improve the robustness of our validation process by ensuring that every molecule in the test set was structurally distinct from those used in the training, closely mimicking a real-world scenario in which a predictive model is often challenged with unprecedented molecular structures. Last, to further test the proposed approach, we only considered cluster-centroids, i.e., a single representative molecule from each cluster, for the training set and singletons in the test set (train: 584 centroids, test: 1,065). In this way, during training, the model was exposed to a wide range of structural motifs but with limited numerosity, further increasing the dissimilarity between training and test compounds.

The Kolmogorov–Smirnov test^35^ applied to the training and test sets based on RDKit fingerprint features confirmed that random splitting preserved feature distributions, with no significantly different bits (0/2048, *p*-value = 0.9975), as expected for this standard approach. Notably, both clustering-based strategies also upheld the IID assumption despite enforcing structural diversity, with all *p*-values well above the conventional 0.05 significance threshold. The clustering-based split exhibited moderate distribution differences (241/2048 bits, *p*-value = 0.7693), whereas the cluster-centroid approach showed minimal differences (7/2048 bits, *p*-value = 0.9236). The lower *p*-value for the clustering-based split aligns with theoretical expectations, as this method imposes stronger structural constraints (see also the Statistical Validation of Splitting Strategies section in SI).

### Model Development and Benchmarking

**Figure 3:**
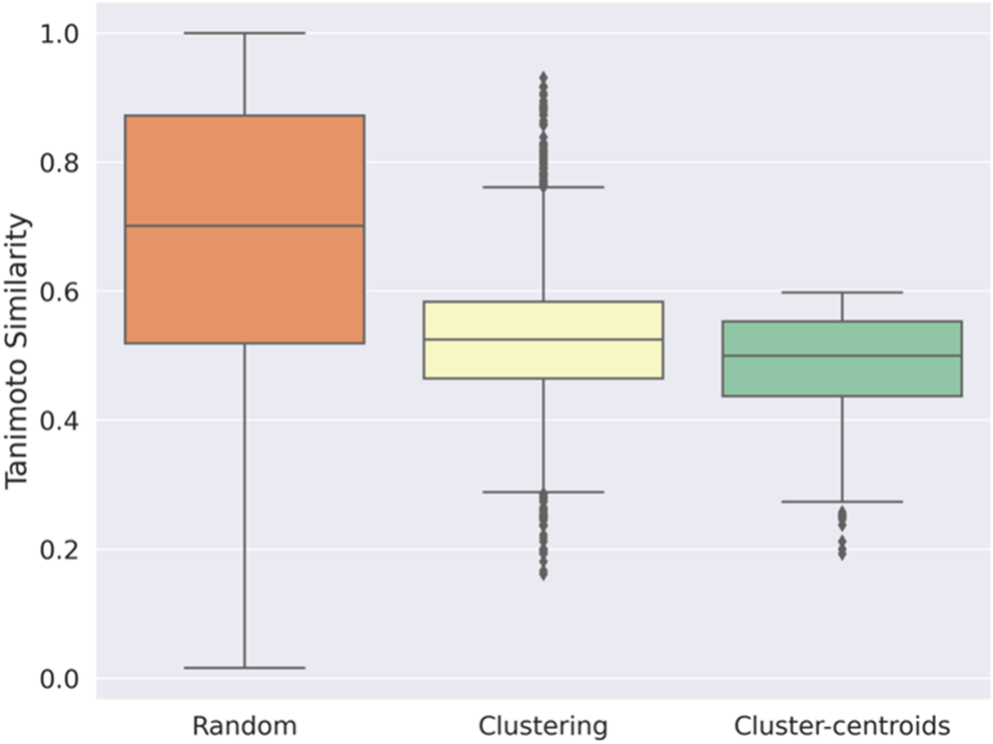
Boxplot displaying the distribution of maximum Tanimoto similarities between training and test sets for different data splitting strategies: random (orange box), clustering (yellow box), and cluster-centroids (green box) splitting.

We initially compared the distributions of maximum Tanimoto similarity between each element of the training and all elements in the test set for the three splitting strategies (Figure 3). The maximum Tanimoto similarity quantifies the similarity between two molecular structures based on their fingerprint representations, where a value of 1 indicates identical sets, and 0 indicates no common elements. Figure 3 shows that the training and test sets were very close from a structural standpoint when data were randomly split (orange box). This significant overlap would likely lead to an unrealistically high performance of the model on the test set that would, however, rapidly degrade on truly unseen data. It could even cause a certain degree of overfitting as the model may focus too heavily on features shared by the two sets, fitting to minor details that are not generalizable. Thanks to clustering, the overlap between test and training sets decreased substantially (yellow box). Switching to cluster-centroids (green box) returned an even greater separation between the structures in the two sets. This provides a more robust assessment of a model’s performance and thus sets an even more realistic expectation on its predicting power. Considering a Tanimoto similarity value of 0.6 as indicative of structural contiguity,^46^ we also added the percentage of maximum similarities that were above the cutoff value for each splitting method. In random splitting, 65% of molecular pairs were above the cutoff, resulting in most of the training and test set pairs being rather similar to one another. With only 18% of pairs being above the cutoff, the clustering splitting strategy had significantly reduced similarities between the sets, proving effective in forcing diversity. Finally, in the cluster-centroid splitting method, no similarity above the cutoff could be identified.

The random, clustering, and cluster-centroids splitting data sets were then used to train and test a Chemprop-based model with 10-fold cross-validation. This software package implements a directed-message-passing neural network (D-MPNN) for molecular property prediction.^22^ Moreover, at this stage, we also included additional 200 molecular features extracted for each compound using RDKit^33^ (see the Model Trainingsection) in order to take into account global properties for each compound in the data set. Having assessed that the models performed similarly on all 10 cross-validation folds, we excluded the presence of bias over a particular subset of data (Table S1). Moreover, we could assume that the performance would have been similar when the models were trained on all data. We then trained the DL-based model across all data sets for random, clustering, and cluster-centroids splitting. Table 2 highlights a trade-off between data set similarity and model performance across AUC, accuracy, recall, and specificity.

**Table 2:** Median Tanimoto Similarities, AUC, Accuracy, Recall, and Specificity for Models Trained on All the Data with Additional RDKit Features for Three Different Splitting Strategies: Random, Clustering, and Cluster-Centroids Splitting

	median Tanimoto similarity	AUC	accuracy	recall	specificity
random	0.70	0.96	0.96	0.98	0.94
clustering	0.52	0.94	0.92	0.94	0.91
cluster-centroids	0.5	0.91	0.90	0.92	0.89

The accuracy of the cluster-centroid-based model (0.90, Table 2) reflects a high overall performance with only a modest drop compared to random splitting. Despite the increased structural diversity between the training and test sets, the model maintains strong accuracy, correctly classifying 90% of compounds. The observed decrease in performance across all metrics is expected, as the cluster-centroid strategy more closely simulates real-world conditions. Nonetheless, the high values (e.g., AUC: 0.91, recall: 0.92) in this more realistic scenario are encouraging. False negatives (missed PBTs) are especially problematic, as these compounds can pose significant environmental risks. Even if displaying high accuracy, a low recall would make the model inadequate for the task at hand. A 0.91 AUC value confirms the model’s discriminative power.^37^

Furthermore, to quantify the impact of the additional RDKit molecular descriptors on training, we also performed parallel training without using the 200 molecular features across all splitting methods as reported by Table S2 in the SI. Performances reported in Table S2 demonstrate that incorporating additional molecular descriptors particularly enhances the model’s predictive capabilities across clustering splitting strategies, likely due to the inclusion of important global molecular properties that complement the local structural information captured by the D-MPNN architecture.^37^ Notably, for the Clustering splitting strategy, the AUC improved from 0.88 to 0.94 when incorporating RDKit molecular descriptors, an approximately 6.4% increase in predictive performance. When the RDKit descriptors were removed, we observed a corresponding performance reduction across different splitting strategies, with the cluster-centroid approach showing a 6.6% decrease in AUC from 0.91 to 0.85. Given these findings, we selected the cluster-centroid-based model with additional RDKit features as the proposed method for PBT prediction, as it provides the most reliable performance on structurally diverse compounds. Also, to place our results in context, we compared the proposed model’s performance with previously published approaches for PBT prediction (Table 3).

**Table 3:** Comparison of Published PBT Prediction Models in Terms of Training Data Set Size, Model Architecture, and Performance Metrics Computed on Different External Validation Sets for Each Study^a^

study	data set size (training)	model type	performance metric
Gramatica and Papa (2010)^16^	180	QSPR MLR model with 4 molecular descriptors	*R*^2^: 80.7%, RMSE_T_: 0.6
Sun et al. (2020)^18^	11,296	DCNN with 2D molecular descriptor matrix	accuracy: 90.4% on REACH PBT assessment list
Wang et al. (2022)^21^	8452	GAT with graph attention networks	accuracy: 94.7%
our study	4104 (80:20 random split)	D-MPNN with RDKit features	accuracy: 96%
	584 (cluster-centroids)	D-MPNN with RDKit features	accuracy: 90%
	584 (cluster-centroids)	D-MPNN	accuracy: 85%

a Model abbreviations: QSPR (quantitative structure–property relationship), MLR (multiple linear regression), DCNN (deep convolutional neural network), GAT (graph attention networks), D-MPNN (directed-message-passing neural network), Metrics: *R*^2^ (coefficient of determination), RMSE (root-mean-square error).

While earlier QSPR approaches demonstrated the feasibility of PBT prediction with 4 molecular descriptors,^16^ modern deep learning methods have achieved superior performance through different implementations. The DCNN approach by Sun et al.^18^ included the largest data set (11,296 chemicals), while the GAT model by Wang et al.^21^ achieved high accuracy using graph attention networks. The D-MPNN-based method adopted in this study attained competitive performance through the effective combination of message-passing and global molecular descriptors.

A quantitative applicability domain analysis was then performed by using two complementary approaches. Using Morgan fingerprints, 276 principal components were required to explain 90% of the variance in the data. The Mahalanobis distances calculated in this space ranged from 5.179 to 23.872 for the training set compounds, with a threshold value (99th percentile) of 23.346. All test compounds fell within this established threshold, indicating 100% coverage. The RDKit descriptor-based analysis required 39 principal components to achieve the same variance coverage. In this space, the Mahalanobis distances for the training set ranged from a minimum value of 2.608 to a maximum of 22.643, with a threshold value (99th percentile) of 13.243. Using this threshold, 6 compounds (0.8%) from the test set exceeded the Mahalanobis distance threshold and were considered outside the applicability domain. These compounds were removed from subsequent analyses, resulting in a final test set of 1059 compounds with validated model applicability (see also the Applicability Domain Analysis section in the SI).

**Figure 4:**
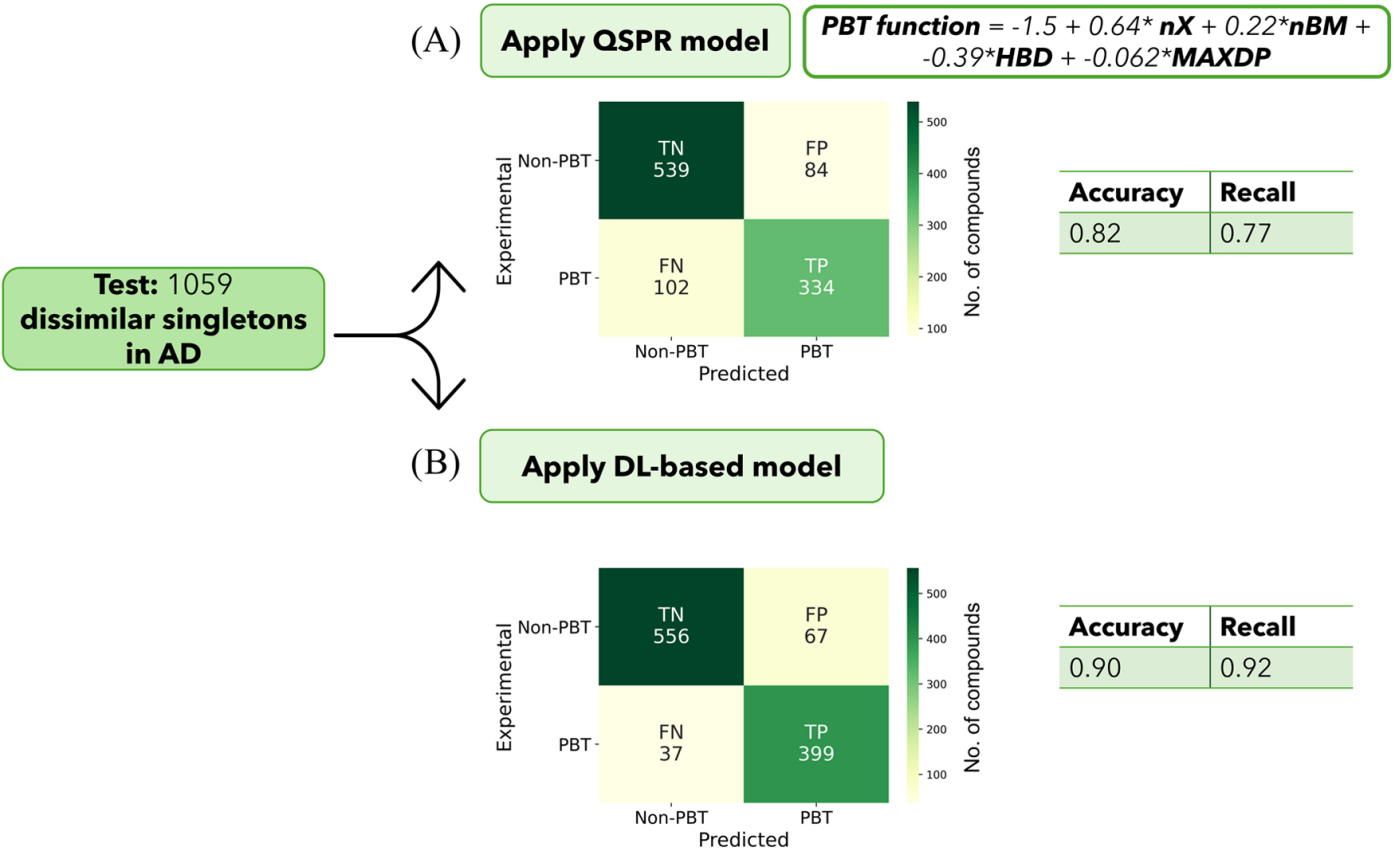
Comparison of GP-QSPR and DL-based model prediction on a test set of 1059 structurally dissimilar compounds within the applicability domain. The confusion matrices for the GP-QSPR model (A) and DL-based model (B) predictions illustrate the performances by extracting accuracy (0.82 vs 0.90) and recall (0.77 vs 0.92) of each model, respectively. The color scale from light yellow to dark green indicates increasing number of compounds in the corresponding quadrant of the confusion matrices.

Finally, we compared the proposed model with the GP-QSPR prediction.^16^ The GP-QSPR model was implemented through a purposely developed Python script (details are reported in the Standard QSPR Model section in the SI) and applied to the test set of 1059 singletons within the proposed model’s applicability domain. The results were expressed as a confusion matrix (Figure 4).

The proposed model outperformed the GP-QSPR model in terms of accuracy and recall. Importantly, the DL-based model was less prone to generating false negatives (FN). As mentioned, this is crucial for any model being applied in real life since these compounds represent a potential safety issue. Future work could involve retraining the original GP-QSPR model on our training set and performing a comparative analysis, provided that experimental data for P, B, and T properties related to these compounds become available.

### Interpretability Analysis and Application to Pharmaceuticals

We analyzed the model’s predictions to identify specific substructures most strongly associated with PBT classification in molecules. Identifying these substructures contributes to explaining how the model makes predictions, ultimately providing actionable knowledge to avoid PBT structural determinants in designing new compounds. The DL-based model leverages a built-in Chemprop function to enhance the explainability. Molecular substructures with more than four atoms that recur and significantly contribute to the PBT classification score are identified and analyzed (see the Model Interpretability and Search for Maximum Common Substructuressection).^41^ We identified 198 substructures considered responsible for the PBT behavior of at least one compound. Of the 18 substructures recurring more than once, 8 could be further grouped after applying a maximum common substructure (MCS) identification procedure, ultimately returning 10 PBT-related substructures and 3 PBT-associated MCSs (Table 4).

**Table 4:**
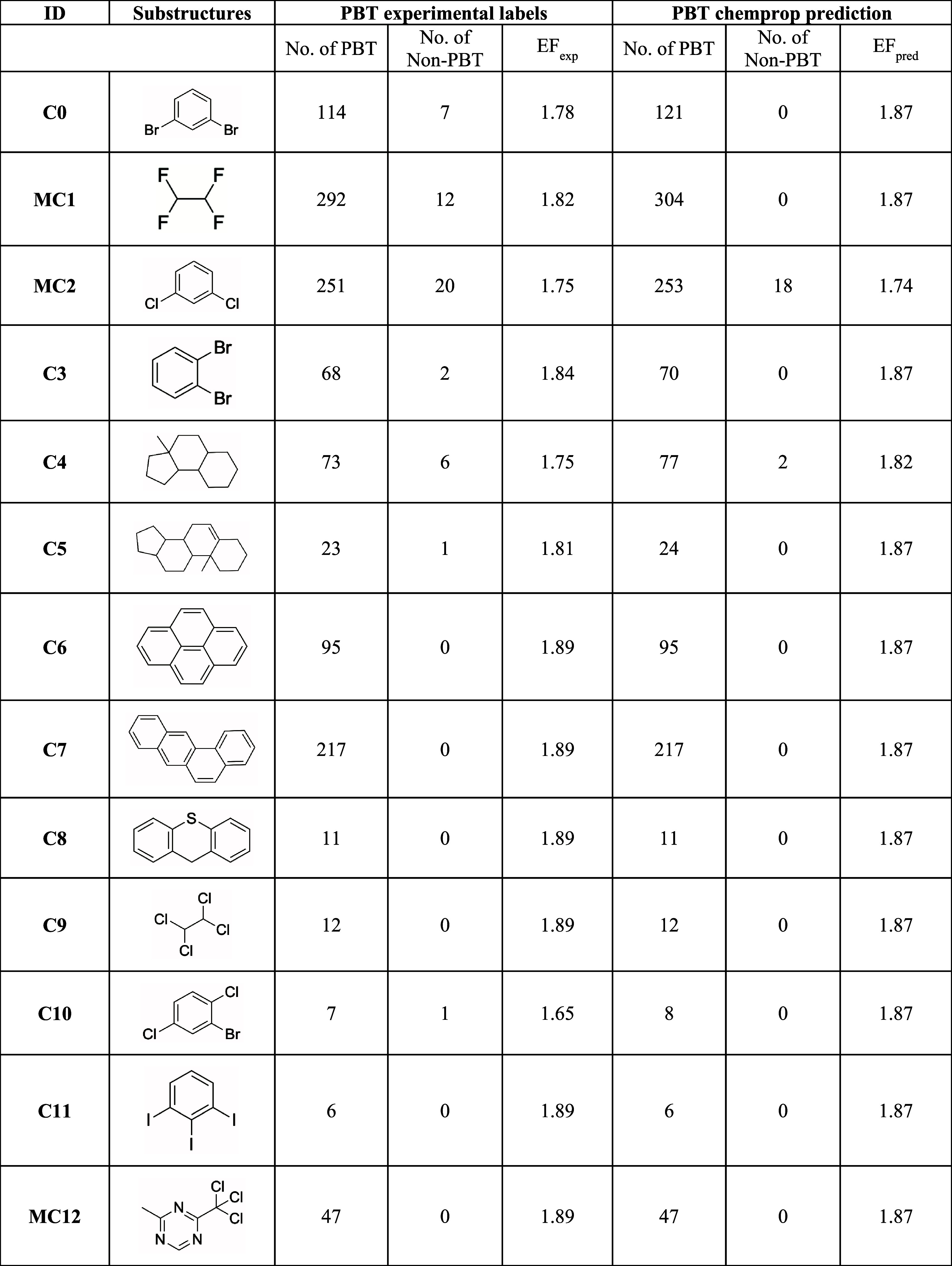
PBT-Relevant Substructures from the Data Set Assembled for This Study^a^

a List of 13 relevant substructures and 3 Maximum Common Substructures (MCSs) present in the curated data set containing 2D structures, matched molecules for PBT and non-PBT profile in the experimental and prediction data set together with the enrichment factor (EF) for the experimental and the prediction data set.

**Figure 5:**
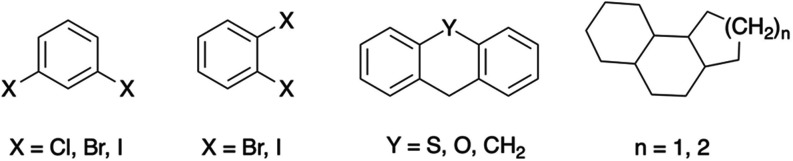
Recurring substructures in the data set that are associated with PBT behavior.

For each substructure, we counted the matching molecules among PBT and non-PBT compounds. In this way, we were able to express the strength of a given substructure’s association to a PBT positive outcome as an enrichment factor (EF). EF values above 1 (Table 4) indicate that the substructure is more often displayed by PBT compounds. Overall, recurrent substructures listed in Table 4 are present in 58% of the compounds categorized as PBT, suggesting a strong correlation between these substructures and PBT behavior. Interestingly, halogen-substituted aromatic rings and tricyclic systems (Figure 5) emerge as structural features that consistently characterize these substructures, in qualitative agreement with the descriptors used in the original GP-QSPR model (eq 1).

**Figure 6:**
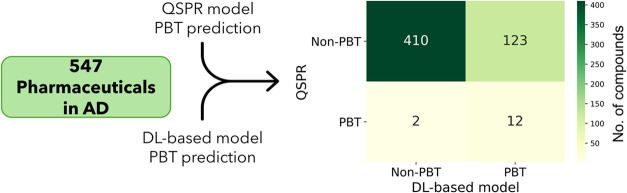
Comparison of QSPR and DL-based model prediction on a data set of 547 pharmaceuticals within the applicability domain. The confusion matrix shows 410 cases where both models agree on non-PBT, 123 cases where QSPR predicts as non-PBT but the proposed model as PBT, 2 cases where QSPR predicts as PBT but the proposed model as non-PBT, and 12 cases where both models agree on PBT. The color scale from light yellow to dark green indicates an increasing number of compounds in the corresponding quadrant of the confusion matrix.

Then, to assess the model’s applicability to other compound classes, we decided to investigate molecules of pharmaceutical interest. Pharmaceuticals have been recently included among the contaminants of emerging concern (CEC).^44^ To this end, we obtained and preprocessed a data set of 559 pharmaceuticals, originally extracted from the DrugBank database, consistently classified as PBT and non-PBT according to two different QSPR models.^44^ The applicability domain analysis showed that all compounds fell within the threshold when using Morgan fingerprints. The RDKit descriptor analysis identified 12 compounds (2.1%) outside the applicability domain After removing these compounds, the consensus classification from the QSPR models was compared with the proposed model’s predictions for the remaining 547 pharmaceuticals (Figure 6).

### Model-Identified PBT Pharmaceuticals and Their Ecological Impact

While the majority of tested compounds were not classified as PBT, the results revealed a nuanced landscape, involving hormones, opioids, antidepressants, and antipsychotics, underscoring a complex and multifaceted effect of contamination by pharmaceuticals in the environment. As reported in Figure 6 (dark green quadrant), 410 pharmaceuticals were unanimously categorized as non-PBT. In contrast, only 12 pharmaceuticals consistently met the PBT criteria. These substances span diverse pharmacological classes, including histaminergic drugs, antifungal agents, hypnotics, anxiolytics, and sedatives (detailed in Table S3). Of particular concern are psychoactive drugs, such as antidepressants and antipsychotics. Several of these compounds have not only been detected in aquatic environments but have also demonstrated the potential to affect aquatic organisms.^47^ A notable example among the PBT-classified compounds is hexachlorophene (HCP). This chlorinated compound, known for its strong bacteriostatic properties, presents significant environmental challenges due to its hydrophobic nature and resistance to biotransformation. Due to a high octanol–water partition coefficient (log *P* = 7.54),^48^ HCP has a strong tendency to bioaccumulate in aquatic organisms. Research indicates that this bioaccumulation can lead to long-term ecological impacts, underscoring the importance of monitoring and managing such persistent compounds in aquatic ecosystems.^49^

Interestingly, the proposed model classified as PBT 123 compounds that the consensus of the previously reported QSPR models labeled as non-PBT. This already points toward the need for investigating further these molecules in an experimental setting for potential PBT/POP properties. For comparison, a distinct data set taken from a study by Howard and Miur was considered.^15^ This data set encompassed 272 chemicals detected in actual environmental media. Of the 12 compounds that both the proposed DL-based model and the QSPR consensus approach classified as PBT (Figure 6, see also Table S3), two could be found in the Howard and Miur data set. The selective serotonin reuptake inhibitor sertraline widely used in the USA since 2018 for the treatment of depression and obsessive compulsive disorder has been detected in surface waters, wastewater treatment plants (WWTP), benthic fauna, and fish tissues with concentrations reaching 17.1 μg/L.^43,50^ Cinnarizine, a histamine receptor subtype H1 antagonist and calcium channel blocker used for the treatment of several conditions including tinnitus and motion sickness, has been detected in surface waters and WWTP sludge.^51^ This molecule is persistent when discharged into the environment, but information regarding its potential for bioaccumulation and ecotoxicity is still lacking.^52^ In the Howard and Miur data set, we could also identify 15 compounds (Table 5) that the proposed model classified as PBT, but which, according to the consensus approach, were not (light green quadrant, Figure 6).

**Table 5:** List of 15 Pharmaceuticals Detected in Environmental Media and Classified as PBT Solely by Chemprop Prediction^a^

CAS	name	pharmaceutical class	ATC code
57-63-6	ethinyl estradiol	estrogen	G03CA01; L02AA03
58-22-0	testosterone	androgen	G03BA03
63-05-8	androstenedione	androgen	
50-28-2	estradiol	estrogen	G03CA03
50-48-6	amitriptyline	antidepressant	N06AA09
50-49-7	imipramine	antidepressant	N06AA02
57-27-2	morphine	opioid	N02AA01
72-33-3	mestranol	estrogen	
76-42-6	oxycodone	opioid	N02AA05
76-57-3	codeine	cough suppressant	R05DA04
76-99-3	methadone	drug used in addictive disorders	N07BC02
125-29-1	hydrocodone	cough suppressants	R05DA03
437-38-7	fentanyl	opioid	N01AH01; N02AB03
19982-08-2	memantine	anti-dementia drug	N06DX01
10238-21-8	glyburide	antidiabetic	A10BB01

a For each compound, the CAS number, name, and pharmaceutical class, linked to the Anatomical Therapeutic Chemical classification (ATC) code, are reported.

These compounds span various pharmacological classes, with hormones, antidepressants, and opioids being the most prevalent. Estrogens and other steroid hormones are known to be persistent and tend to be absorbed in soil and sediments, bioaccumulating in the environment.^51^ In particular, ethinyl estradiol, a semisynthetic, orally administered estradiol analogue, is considered an endocrine-disrupting chemical (EDC) and it has been extensively studied for its environmental impact. It is persistent in the environment, bioaccumulative, and toxic to aquatic life.^53^ It is known to cause endocrine disruption in fish at very low concentrations with a predicted no-effect concentration (PNEC) of 0.035 ng/L.^53^ Opioids, including morphine, codeine, and their derivatives, were also identified by our analysis. These compounds have been detected in surface waters, with concentrations ranging from ng/L to μg/L raising concerns about their potential ecological impact.^54^ It should be pointed out that certain psychoactive substances can exhibit pseudopersistence due to their continuous input into aquatic environments.^55^ A study from Ding and Zhang has shown that psychoactive compounds can bioaccumulate in the tissues and organs of aquatic organisms, potentially leading to acute or chronic toxic effects.^55^ For instance, morphine, detected in surface water at concentrations up to 148 ng/L, has been found to impact the immune system of freshwater mussels, potentially affecting the entire ecosystem.^54^ Antidepressants and antipsychotics account for a significant share of compounds reported in Table 5, with drugs like amitriptyline, imipramine, and paroxetine associated with varying degrees of environmental persistence. Amitriptyline, a tricyclic antidepressant (TCA), has been detected in surface waters at concentrations ranging from 20 to 26 ng/L, as was also found in WWTP effluents in France at 6.0 ng/L.^47^ Its nonbiodegradability under sewage treatment conditions suggests a potential for environmental persistence. Similarly, imipramine and paroxetine exhibit persistence in aquatic environment due to their chemical stability and resistance to degradation processes.^47^ Even at low concentrations, they can induce behavioral changes in fish and other aquatic species, potentially affecting population dynamics and ecosystem function.^47^ Many of the identified compounds display high lipophilicity; this feature often correlates with a higher potential for bioaccumulation in aquatic organisms and persistence in sediments, further amplifying their potential environmental impact.^47^ Lastly, among the 410 pharmaceuticals recognized as non-PBT by both predictions, 55 chemicals were found in the environment according to Howard and Muir.^15^ Among those, antibiotics represent a significant class of concern defined as major pollutants in aquatic environments.^56^ Ciprofloxacin, for example, demonstrates strong persistence through soil adsorption and sediment accumulation, with concentrations reaching up to 31,000 ng/L in hospital effluents and showing notable toxicity to cyanobacteria even at low concentrations.^56^ The sulfonamide Sulfamethoxazole, commonly detected in surface waters at concentrations ranging from 0.1 ng/L to 532 ng/L, has been shown to significantly affect algal growth at environmental concentrations. Erythromycin exhibits considerable environmental stability under various conditions, with detected concentrations ranging from 2.2 to 54.8 ng/L in groundwater, and shows potential for bioaccumulation in aquatic organisms.^56^ Pharmaceuticals classified as non-PBT through predictive models may still exhibit concerning environmental behaviors and impacts when released into natural systems. One possible explanation on why several predictive attempts, including ours, struggled at correctly labeling these molecules could be that these compounds might be persistent or bioaccumulative but not toxic.^15^ This hypothesis is supported by studies showing that many pharmaceuticals, while not acutely toxic, can persist in the environment for extended periods or bioaccumulate in organisms without showing immediate toxic effects.^57^ For instance, as reported in a recent review, some antibiotics can persist in sediments for over 100 days and accumulate in aquatic organisms at levels above environmental concentrations,^56^ yet may not trigger traditional toxicity end points in standard assessment protocols.^57^ We suggest that future efforts are focused on reevaluating these compounds to better understand their environmental impact. With these models, such as this proposed, they can be further improved and potentially become more useful in focusing PBT testing efforts on compounds at earlier stages in drug discovery or other novel chemical discovery processes.

### Identification of PBT-Related Substructures on Pharmaceuticals

We also investigated the presence of the previously identified PBT-related substructures in the pharmaceuticals included in the data set. Results are reported in Table 6, with commercial drugs detected in environmental media according to Howard and Miur highlighted.^44^

**Table 6:**
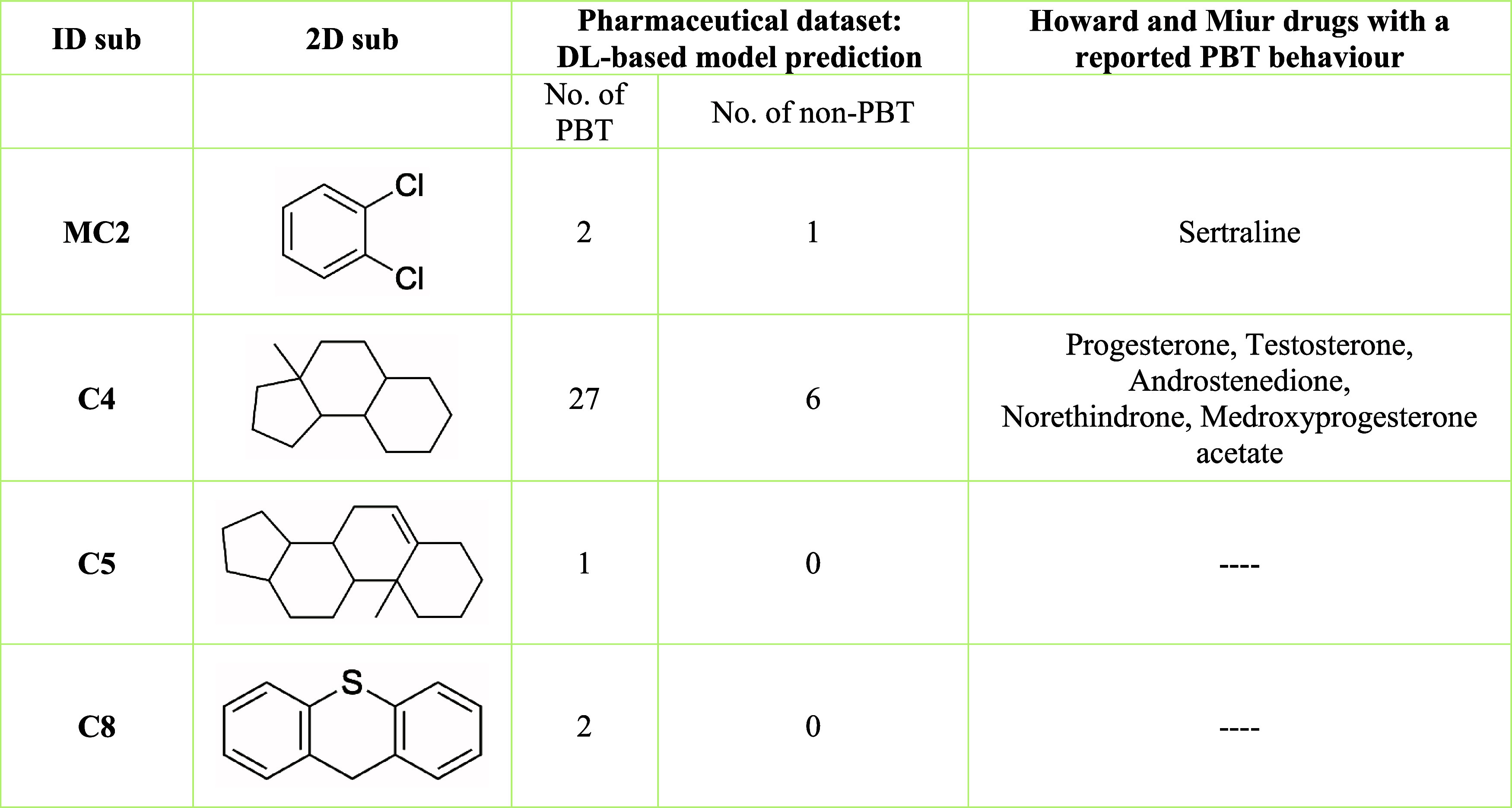
List of PBT-Relevant Substructures Identified in the Pharmaceutical Data Set^a^

a For each substructure, the matched number of molecules in the pharmaceutical data set for PBT and non-PBT, together with names of matched molecules detected in environmental media with PBT positive behavior according to Howard and Miur, are reported.

Tricyclic substructures such as C4, C5, and C8 (Table 6) are particularly prevalent among PBT positive pharmaceutical molecules. Molecules bearing the C4 substructure, which is associated with steroid hormones, are predominantly classified as PBT (27 PBT vs 6 non-PBT). This aligns with the already mentioned environmental persistence of sex hormones and their tendency to bioaccumulate in aquatic environments.^51^ Substructure MC2, characterized by a halogen-substituted aromatic ring, was also associated with PBT positive behavior. Notably, sertraline displays substructure MC2, suggesting how this substructure could lead to environmental persistence. This substructure analysis reveals that specific structural features linked to PBT behavior, originally identified in a data set of general-purpose chemicals, are commonly present in commercial drugs.

Then, using Chemprop’s built-in interpret function (see the Model Interpretability and Search for Maximum Common Substructuressection), we extracted PBT-associated substructures from the data set of pharmaceutical compounds. We identified 85 substructures associated with a PBT label, with 10 appearing more than once as reported in Table S4. PC0 and PC8 overlap with structures in Table 6, confirming their role in assigning a PBT positive prediction. The role of tricyclic substructures (Figure 5) emerged once again as conducive to PBT positive behavior. Interestingly, PC2 and PC3 are associated with opioids, like morphine, codeine or oxycodone, and hydrocodone, previously reported as environmental contaminats.^54^

In 2004, Kola and Landis highlighted how poor pharmacokinetics (PK) and unforeseen toxicity were among the major contributors to high attrition rates in drug development.^58^ They proposed that avoiding specific chemical substructures known to cause PK and toxicity issues and addressing these elements as early as lead optimization could reduce late-stage failures. By ingraining predictive strategies early in the design process, compounds likely to fail due to PK or toxicity issues could be discarded, thereby saving significant time and resources. As environmental impact concerns regarding pharmaceuticals continue to grow, this principle of early-stage risk prediction can be extended beyond the PK and toxicity to encompass environmental factors. Similar to how certain substructures are linked to adverse PK profiles and toxicity, in this study, a straightforward way to extract chemical features associated with environmental persistence and bioaccumulation, potentially leading to ecological harm is presented.^59^ An easy-to-update collection of PBT-related substructures can be used to flag and, possibly, filter out potential pollutants, improving drug sustainability and reducing downstream attrition.

## Conclusions

In this study, we developed a predictive model for classifying compounds as PBT or non-PBT. By employing clustering techniques to enhance the structural diversity between training and test sets, we ensured a realistic evaluation of the model’s performance, avoiding artificially inflated results. The model achieved high accuracy and interpretability, effectively identifying structural features associated with PBT behavior. Notably, it flagged potential PBT pharmaceuticals, some of which have been independently verified in environmental studies. Despite its strengths, the model has limitations. As a classification tool, it provides binary outputs (PBT or non-PBT) rather than detailed predictions about the extent or nature of a compound’s persistence, bioaccumulation, or toxicity. Our preprocessing approach follows common cheminformatics practices regarding salt removal, as certain inorganic compounds due to technical limitations cannot be effectively converted into the molecular graphs required for the deep learning method adopted here. Future work should consider developing methods that can appropriately account for the contribution of both organic and inorganic components to PBT properties, potentially through the development of hybrid modeling approaches that can handle both classical organic structural features and metal-specific parameters.

We also identified key PBT-relevant substructures that could serve as structural flags. These substructures are particularly valuable for rapidly screening large chemical libraries and guiding early-stage compound design to avoid the development of environmentally persistent pollutants.

The model’s utility and the comprehensiveness of the substructure library can only be improved with access to more robust and standardized data on the PBT behavior of pharmaceuticals. Currently, inconsistencies in measuring and reporting end points for persistence, bioaccumulation, and toxicity present significant challenges.^32^ Standardized protocols—such as agreed-upon half-lives in environmental matrices, bioconcentration factors across relevant species, and cross-trophic level toxicity end points—are urgently needed to provide the foundation for more precise models.^60,61^

Looking forward, combining substructure filters for rapid screening with detailed predictive models for nuanced assessments could represent an efficient strategy for PBT evaluation in drug development. Moreover, our model could be integrated with existing tools to enhance confidence in PBT predictions through consensus. With expanded data sets and standardized experimental methods, future research could refine predictions, expand applicability to diverse species and chronic exposures, and incorporate mixture toxicity. While the model described here offers a valuable early tool for PBT classification, it highlights the critical need for high-quality, standardized data to drive the next generation of predictive modeling in this field.

## Data Availability

We freely provide all the data sets used in this work, outputs, as well as Jupyter Notebooks to reproduce our results and the figures reported in this work at https://github.com/domingasbd/PBT-project. At the same link, we also provide a Jupyter Notebook with guidance on how to make predictions of PBT behavior using the proposed DL-based model on other data sets.

## Supporting Information Available

Brief description of data sets used in the work; standard QSPR Model: details on a Python script developed to calculate the PBT function according to the original QSPR equation, t-SNE plot: Information on how t-SNE was applied to visualize the data, Butina clustering: description of the splitting strategy used for clustering compounds, statistical validation of splitting strategies, threshold selection analysis for binary classification; distribution of the proposed model prediction scores for training and test sets (Figure S2); training set and test set accuracies across different probability thresholds (Figure S3); performance metrics (AUC, accuracy, recall, specificity) for different data splitting strategies obtained after 10-fold cross-validation models with additional RDKit features (Table S1); performance metrics (AUC, accuracy, recall, specificity) for different data splitting strategies obtained after models trained on all the data without additional RDkit features (Table S2); 12 pharmaceuticals consistently classified as PBT by both QSAR predictions and the deep learning-based model (Table S3); and PBT-relevant substructures extracted from pharmaceuticals (Table S4) (PDF)
