# Supplementary material for: Application of Deep Learning to Predict the Persistence, Bioaccumulation, and Toxicity of Pharmaceuticals
Source: J Chem Inf Model. 2025 Apr 3;65(7):3248–61. doi: 10.1021/acs.jcim.4c02293 (PMC12004513; doi:10.1021/acs.jcim.4c02293)
Supplement: Supplementary file 1 — ci4c02293_si_001.pdf [file ci4c02293_si_001.pdf]

SUPPORTING INFORMATION

for

Application of Deep Learning to predict the Persistence, Bioaccumulation, and Toxicity of Pharmaceuticals

Dominga Evangelista,[1] Elliot Nelson,[2] Rachel Skyner,[2] Ben Tehan,[2] Mattia Bernetti,[3,4] Marinella Roberti,[1] Maria Laura Bolognesi,[1,*] and Giovanni Bottegoni [3,5,*]

TABLE OF CONTENTS

DATASETS 3

PAPA AND GRAMATICA. 3
DATASET PURPOSELY ASSEMBLED FOR THIS STUDY. 3
AGROCHEMICALS. 3
DRUGBANK. 3
PHARMACEUTICALS. 3
HOWARD AND MIUR. 3

STANDARD QSPR MODEL 3

T-SNE DIMENSIONALITY REDUCTION ANALYSIS 4

FIGURE S1 4

BUTINA CLUSTERING: SPLITTING STRATEGY 4

STATISTICAL VALIDATION OF SPLITTING STRATEGIES 5

THRESHOLD SELECTION ANALYSIS FOR BINARY CLASSIFICATION 5

FIGURE S2 5
FIGURE S3 6

APPLICABILITY DOMAIN ANALYSIS 6

PERFORMANCE METRICS AND MODELS EVALUATION 7

TABLE S1 7
TABLE S2 7
TABLE S3: PHARMACEUTICALS CONSISTENTLY CLASSIFIED AS PBT. 8
TABLE S4: PBT-RELEVANT SUBSTRUCTURES FROM PHARMACEUTICALS. 8

REFERENCES 10

Datasets

A short description of each dataset used in this work, as listed in Table 1 of the main text, is reported.

Papa and Gramatica. 250 heterogeneous compounds were retrieved from Papa and Gramatica[1] with experimental data for all three properties: Persistence (P) measured in terms of Global Half-Life Index (GHLI); Bio-accumulation (B) measured in terms of logBCF; and Toxicity (T) measured in terms of Experimental 96h LC_{50}.[1]

Dataset purposely assembled for this study. 6072 molecules were initially retrieved: 2970 identified non-Persistent Organic Pollutants (POPs) and 3102 validated Persistent Bioaccumulative Toxic (PBT) or POPs chemicals. The latter group included 2785 potential PBT chemicals identified by Strempel et al.[2] and 317 expert-verified PBT chemicals from the European Chemicals Agency (ECHA) PBT/vPvB assessments, the ECHA PBT assessment list, the ECHA list of substances subject to POP Regulation, and the new POP list under the Stockholm Convention.[3] The 2970 Non-PBT chemicals included 2887 compounds from the ECHA-registered substances, 48 expert-verified compounds gathered from the ECHA PBT/vPvB assessments, and 35 compounds from the ECHA PBT assessment list.[4]

Agrochemicals. The Pesticide Property Database[5] (PPDB) encompasses 1779 agrochemicals. The standardized sanitization process reported below was applied preserving stereochemistry details. A dataset containing 1277 agrochemicals was obtained.

Drugbank. 11915 drugs from the DrugBank database[6] were collected and standardized with the procedure described below preserving stereochemical details. The final DrugBank dataset encompassed 11168 drugs.

Pharmaceuticals. 619 drugs originally screened and labelled according to the consensus of two QSAR models (Insubria-PBT and US-EPA PBT profiler) by Sangion and Gramatica were retrieved from DrugBank.[6, 7] Molecules were standardized with the procedure described below preserving stereochemical details. The dataset of molecules of pharmaceutical interest eventually encompassed 559 drugs, 544 labelled as non-PBT and 15 as PBT.[7]

Howard and Miur. 277 chemicals annotated by name and CAS number were retrieved from a study by Howard and Miur reporting compounds detected in environmental media.[8]

Standard QSPR Model

A Python script was developed to calculate the PBT function (1) according to the original QSPR equation developed by Papa and Gramatica using experimental data for P, B and T.

$$(1)\quad PBT\ function = -1.5 + 0.64 * nX + 0.22*nBM + -0.39*HBD + -0.062*MAXDP$$

This script takes a SMILES string as input and uses RDKit[9] modules to extract from each compound: nX, the number of halogens; HBD, the number of hydrogen bond donors according to Lipinski; nBM, the number of multiple bonds; and MAXDP, the maximum electropological positive variation. Equation (1) is solved plugging in the extracted values and a PBT value is returned.

t-SNE Dimensionality Reduction Analysis

The sklearn.manifold's t-distributed stochastic neighbor embedding (t-SNE) function was applied through a purposely developed Python script. Each molecule was annotated according to 200 RDKit molecular descriptors encompassing a broad range of molecular properties including topological features (like atom and bond counts, molecular connectivity indices), physicochemical properties (such as calculated logP, molecular weight, polar surface area), geometric characteristics (ring counts, stereocenters), and electronic parameters. This comprehensive set of descriptors provides a robust characterization of the molecular space and corresponds directly to the features used to enrich our model training. The t-SNE analysis reduces this high-dimensional data to a two-dimensional representation while preserving associations between data points.

Figure S1 reveals the chemical space distribution between pharmaceutical compounds from DrugBank (pink) and agrochemical (yellow).

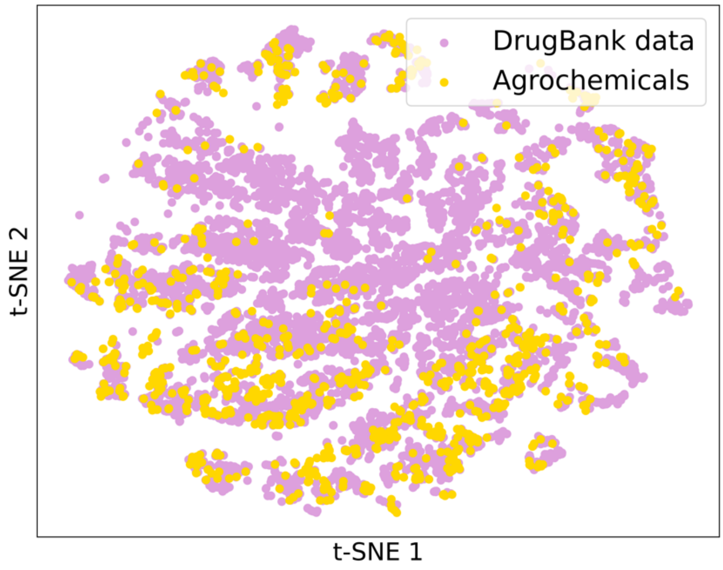

Figure S1: **t**-SNE plot representing high-dimensional molecular data in two dimensions with 200 RDKit molecular features mapping the overlap between DrugBank data (pink) and Agrochemicals (yellow) in the defined chemical space.

Butina Clustering: Splitting Strategy

The procedure developed by Sidow and colleagues[10] to perform clustering according to the algorithm described by Butina[11] was employed. To identify chemical similarities, molecules were represented as subgraph-based RDKit fingerprints.[9] Tanimoto distance was used to express the distance between fingerprints. The cut-off value for clustering was set at 0.4. This cut-off value was chosen because it provides a good balance between identifying a meaningful partition (1650 clusters) and avoiding too many singletons (1065).

Statistical Validation of Splitting Strategies

The "Independent and Identically Distributed" (IID) assumption is standard for machine learning methods. To evaluate whether the splitting strategies adopted in this study preserved this assumption, a Kolmogorov-Smirnov (KS)[12] test was performed on RDKit fingerprint features. The KS test[13] compares the empirical distribution functions of two samples by calculating the maximum distance between the cumulative distribution functions (KS statistic D) and estimate the probability of observing such differences if samples were drawn from the same distribution.
For each splitting strategy, RDKit fingerprints (2048 bits) were generated for all compounds, and the distribution of each fingerprint bit was compared between training and test sets using a significance level of 0.05. The random splitting approach showed perfect preservation of feature distributions, with no significantly different bits and a p-value of 0.9975 (mean KS statistic = 0.0082). The clustering-based splitting demonstrated moderate distribution differences with 241 significantly different bits out of 2048 (11.8%) and a p-value of 0.7693 (mean KS statistic = 0.0321). The cluster-centroids splitting achieved minimal distribution differences with only 7 significantly different bits (0.3%) and a p-value of 0.9236 (mean KS statistic = 0.0175). P-values >0.05 across all strategies indicate preservation of the IID assumption, supporting the validity of all splitting approaches.

Threshold Selection Analysis for Binary Classification

The optimal classification threshold was determined for the final cluster-centroids model (training: n=584 centroids; test: n=1065 singletons). Analyses for other splitting strategies are available at https://github.com/domingasbd/PBT-project.

The distribution of prediction scores was analyzed in Figure S2, revealing bimodal patterns across training and test sets with, as expected, peaks near 0 and 1 The optimal threshold of 0.38, selected based on maximum training accuracy, falls within a stable performance region (0.2-0.6) where the model achieves accuracies of 0.96 and 0.90 for training and test sets respectively (Figure S3).

Figure S2 reveals the distribution of model prediction scores for training and test set for the cluster-centroids model.

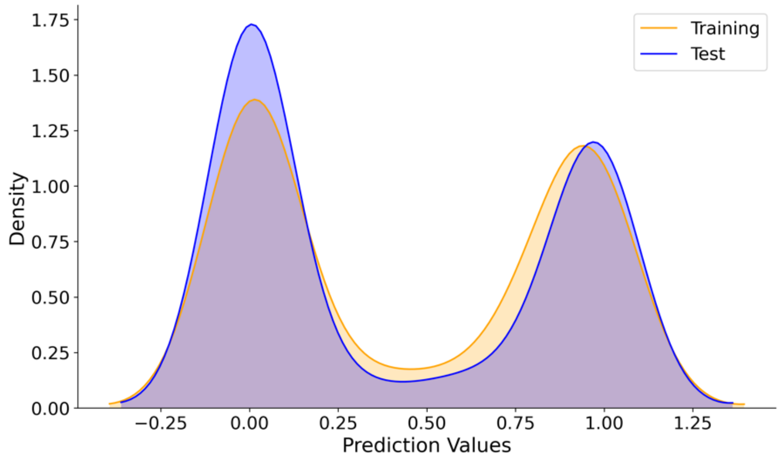

Figure S2: *Distribution of Model Prediction Scores for Training and Test Sets.*

Figure S3 reveals the training and test set accuracies across different probability thresholds for the cluster-centroids model.

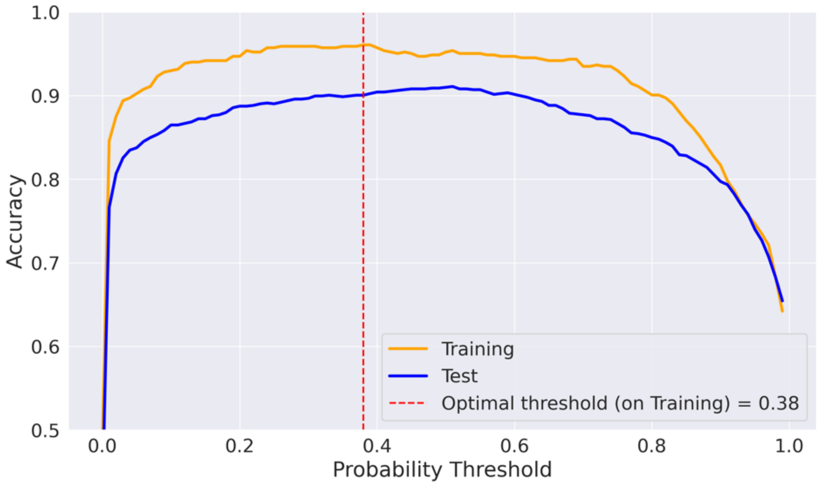

Figure S3: *Training and Test Set Accuracies Across Different Probability Thresholds. The optimal threshold (0.38, vertical red line) was selected based on training set accuracy. Both training (orange line) and test set (blue line) accuracies show stable performance across a broad range of threshold values (0.2-0.6), indicating robust model generalization.*

Applicability Domain Analysis

The applicability domain (AD) analysis was performed using two complementary approaches: Morgan fingerprints (radius=2 and 2048 bits) and 200 RDKit molecular descriptors. For both approaches, molecular features were first scaled using min-max normalization to ensure equal feature weighting. The AD was established through a combined Principal Component Analysis (PCA)[14] and Mahalanobis distance method, using the training set of cluster centroids (n=584) as the reference space.

Principal Component Analysis was employed to reduce the dimensionality of the molecular representation while preserving 90% of the cumulative variance. For Morgan fingerprints, this required 276 principal components, reflecting the high-dimensional nature of the fingerprint representation. The RDKit descriptor approach required 39 principal components to explain the 90 % of the variance.

The Mahalanobis distance was then calculated in the PCA-transformed space to account for the correlation between variables and the different scales of variance along principal component axes. For a compound with feature vector x, the Mahalanobis distance d to the training set centroid μ was computed as:

d = sqrt((x - μ)^T S^-1 (x - μ))

where S is the covariance matrix of the training set in the PCA space. The AD threshold was established at the 99th percentile of the training set distances, with compounds exceeding this threshold considered outside the model's AD. This threshold choice provides a balance between being too restrictive and too permissive in defining the AD boundaries.

Performance Metrics and Models Evaluation

Table S1 reports the mean and the standard deviation for AUC, Accuracy, Recall and Specificity for each of the three splits of the data: random splitting, clustering splitting and cluster-centroids splitting. Performance metrics in terms of AUC, Accuracy, Recall and Specificity were obtained after training 10-fold cross-validation models also enhancing input compounds with 200 molecular features.

	AUC		Accuracy		Recall		Specificity	
	**Mean**	**SD**	**Mean**	**SD**	**Mean**	**SD**	**Mean**	**SD**
Random splitting	0.991	0.002	0.982	0.012	0.990	0.009	0.973	0.016
Clustering splitting	0.976	0.001	0.986	0.006	0.992	0.006	0.978	0.008
Cluster-centroids splitting	0.951	0.003	0.964	0.026	0.970	0.046	0.959	0.034

Table S1: *Mean and Standard Deviation Results for AUC, Accuracy, Recall and Specificity of Cross-validation models. Low standard deviation values for AUC, Accuracy, Recall and Specificity imply that the models perform similarly on all folds, suggesting the absence of bias over a particular subset of data.*

Table S2 reports AUC, Accuracy, Recall and Specificity for models trained on all the data without additional RDkit features for three different splitting strategies: random, clustering and cluster-centroids splitting.

	AUC	Accuracy	Recall	Specificity
Random	0.94	0.94	0.95	0.93
Clustering	0.88	0.89	0.82	0.95
Cluster-centroids	0.85	0.85	0.87	0.83

Table S2: AUC, Accuracy, Recall and Specificity for models trained on all the data without additional RDkit features for three different splitting strategies

To evaluate the impact of the additional RDKit molecular descriptors, we also performed parallel training without the 200 molecular features across all splitting methods as shown by Table S2. When comparing the performances of models trained on the complete dataset without additional RDkit features, interesting patterns emerged. For random splitting, both approaches achieved comparable high performance, with the model with additional features showing similar results (Table 2, AUC: 0.96, Accuracy: 0.96, Recall: 0.98, Specificity: 0.94) to the model without additional features (Table S2, AUC: 0.94, Accuracy: 0.94, Recall: 0.95, Specificity: 0.93). However, the impact of additional features became more pronounced in clustering-based approaches. In clustering splitting, the model with additional features (Table 2, AUC: 0.94, Accuracy: 0.94, Recall: 0.93, Specificity: 0.94) significantly outperformed the model without them (Table S2, AUC: 0.88, Accuracy: 0.89, Recall: 0.82, Specificity: 0.95). Similarly, for cluster-centroids splitting, the inclusion of RDKit features yielded better results (Table 2, AUC: 0.91, Accuracy: 0.91, Recall: 0.90, Specificity: 0.92) compared

to the model without them (Table S2, AUC: 0.85, Accuracy: 0.85, Recall: 0.87, Specificity: 0.83). By incorporating molecular descriptors alongside the D-MPNN's local structural training, the model provides better predictions on both clustering-based approaches.

Table S3: Pharmaceuticals Consistently Classified as PBT.

The 12 pharmaceuticals consistently classified as PBT by the previously reported consensus of QSAR predictions and the DL-based model (Figure 6 in the main text), annotated by CAS number, common name, pharmaceutical class linked to the Anatomical Therapeutic Chemical (ATC) classification code, are reported in Table S5. Out of these 12 compounds, Sertraline and Cinnarizine could be found in the Howard and Miur dataset as molecules detected in environmental media.

CAS	Name	Pharmaceutical class	ATC code
52468-60-7	Flunarizine	Calcium antagonist	N07CA03
53179-11-6	Loperamide	Antidiarrheal	A07DA03
79617-96-2	Sertraline	Psychoactive	N06AB06
50-53-3	Chloropromazine	Psychoactive	N05AA01
70-30-4	Hexachlorophene	Antiseptic	D08AE01
97-18-7	Bithionol	Anthelmintic	D10AB01<br>P02BX01
113-59-7	Chlorprothixene	Psychoactive	N05AF03
298-57-7	Cinnarizine	Antihistaminic	N07CA02
303-49-1	Clomipramine	Psychoactive	N06AA04
911-45-5	Clomiphene	Estrogen agonist	G03GB02
1841-19-6	Fluspirilene	Psychoactive	N05AG01
2398-96-1	Tolnaftate	Antifungal agent	D01AE18

Table S3: list of 12 pharmaceuticals classified as PBT by the consensus of QSAR predictions and Chemprop prediction: CAS, name, Pharmaceutical class linked to Anatomical Therapeutic Chemical classification code (ATC) code.

Table S4: PBT-relevant substructures from pharmaceuticals. Of the 86 substructures associated with a PBT-positive label and identified thanks to the procedure described in the model interpretability section, 10 repeated more than once.

**ID**	**Substructure**	**Pharmaceutical dataset DL-based model prediction**		**PBT compounds from Howard and Miur**
		N. of PBT	N. of non-PBT	
**PC0**	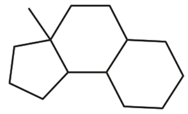	27	6	Testosterone, Androstenedone
**PC1**	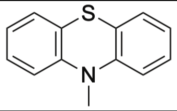	13	0	----

**PC2**	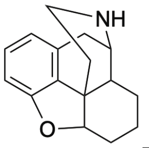	12	0	Oxycodone, Hydrocodone
**PC3**	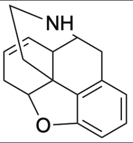	6	0	Morphine, Codeine
**PC4**	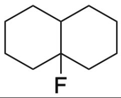	4	0	----
**PC5**	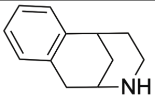	15	0	Oxycodone, Hydrocodone
**PC6**	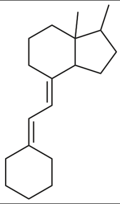	2	0	----
**PC7**	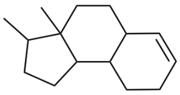	0	0	----
**PC8**	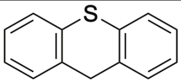	2	0	----
**PC9**	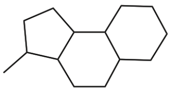	18	5	----

Table S4: molecules labelled as PBT and Non-PBT bearing PBT-related substructures. Molecules experimentally detected in environmental media with confirmed PBT positive behavior are named explicitly.

References

(1) Papa, E.; Gramatica, P. QSPR as a Support for the EU REACH Regulation and Rational Design of Environmentally Safer Chemicals: PBT Identification from Molecular Structure. *Green Chemistry* **2010**, *12* (5), 836–884. https://doi.org/10.1039/b923843c.

(2) Scheringer, M.; Strempel, S.; Ng, C. A.; Hungerbühler, K. Response to Comment on Screening for PBT Chemicals among the "Existing" and "New" Chemicals of the EU. *Environ Sci Technol* **2013**, *47* (11), 6065–6066. https://doi.org/10.1021/es401769z.

(3) No Title. https://pops.int/.

(4) Legislation @ Echa.Europa.Eu.

(5) Lewis, K. A.; Tzilivakis, J.; Warner, D. J.; Green, A. An International Database for Pesticide Risk Assessments and Management. *Human and Ecological Risk Assessment* **2016**, *22* (4), 1050–1064. https://doi.org/10.1080/10807039.2015.1133242.

(6) Wishart, D. S.; Knox, C.; Guo, A. C.; Cheng, D.; Shrivastava, S.; Tzur, D.; Gautam, B.; Hassanali, M. DrugBank: A Knowledgebase for Drugs, Drug Actions and Drug Targets. *Nucleic Acids Res* **2008**, *36* (SUPPL. 1), 901–906. https://doi.org/10.1093/nar/gkm958.

(7) Sangion, A.; Gramatica, P. PBT Assessment and Prioritization of Contaminants of Emerging Concern: Pharmaceuticals. *Environ Res* **2016**, *147*, 297–306. https://doi.org/10.1016/j.envres.2016.02.021.

(8) Howard, P. H.; Muir, D. C. G. Identifying New Persistent and Bioaccumulative Organics among Chemicals in Commerce. III: Byproducts, Impurities, and Transformation Products. *Environ Sci Technol* **2013**, *47* (10), 5259–5266. https://doi.org/10.1021/es4004075.

(9) Bento, A. P.; Hersey, A.; Félix, E.; Landrum, G.; Gaulton, A.; Atkinson, F.; Bellis, L. J.; De Veij, M.; Leach, A. R. An Open Source Chemical Structure Curation Pipeline Using RDKit. *J Cheminform* **2020**, *12* (1), 1–16. https://doi.org/10.1186/s13321-020-00456-1.

(10) Sydow, D.; Morger, A.; Driller, M.; Volkamer, A. TeachopenCadd: A Teaching Platform for Computer-Aided Drug Design Using Open Source Packages and Data. *J Cheminform* **2019**, *11* (1), 1–7. https://doi.org/10.1186/s13321-019-0351-x.

(11) Butina, D. Unsupervised Data Base Clustering Based on Daylight's Fingerprint and Tanimoto Similarity: A Fast and Automated Way to Cluster Small and Large Data Sets. *J Chem Inf Comput Sci* **1999**, *39* (4), 747–750. https://doi.org/10.1021/ci9803381.

(12) Pratt, J. W.; Gibbons, J. D. Kolmogorov-Smirnov Two-Sample Tests. **1981**, 318–344. https://doi.org/10.1007/978-1-4612-5931-2_7.

(13) Kim, S. H.; Whitt, W. The Power of Alternative Kolmogorov-Smirnov Tests Based on Transformations of the Data. *ACM Transactions on Modeling and Computer Simulation* **2015**, *25* (4). https://doi.org/10.1145/2699716/SUPPL_FILE/KIM.ZIP.

(14) Qin, L. T.; Zhang, J. Y.; Nong, Q. Y.; Xu, X. C. L.; Zeng, H. H.; Liang, Y. P.; Mo, L. Y. Classification and Regression Machine Learning Models for Predicting the Combined Toxicity and Interactions of Antibiotics and Fungicides Mixtures. *Environmental Pollution* **2024**, *360*, 124565. https://doi.org/10.1016/J.ENVPOL.2024.124565.
